# Inflammation-Mediated Regulation of MicroRNA Expression in Transplanted Pancreatic Islets

**DOI:** 10.1155/2012/723614

**Published:** 2012-05-10

**Authors:** Valia Bravo-Egana, Samuel Rosero, Dagmar Klein, Zhijie Jiang, Nancy Vargas, Nicholas Tsinoremas, Marco Doni, Michele Podetta, Camillo Ricordi, R. Damaris Molano, Antonello Pileggi, Ricardo L. Pastori

**Affiliations:** ^1^Diabetes Research Institute, University of Miami, Miami, FL, USA; ^2^Center for Computational Science, University of Miami, Miami, FL, USA; ^3^DeWitt Daughtry Department of Surgery, University of Miami Leonard M. Miller School of Medicine, Miami, FL, USA; ^4^Department of Medicine, University of Miami Leonard M. Miller School of Medicine, Miami, FL, USA; ^5^Institute of Hepatopancreatic Surgery, Istituto di Ricovero e Cura a Carattere Scientifico, Fondazione Policlinico San Matteo, Pavia, Italy; ^6^Department of Surgical Sciences, University of Pavia, Pavia, Italy; ^7^Department of Microbiology and Immunology, and Department of Medicine, University of Miami Leonard M. Miller School of Medicine, Miami, FL, USA; ^8^Department of Biomedical Engineering, University of Miami, Miami, FL, USA

## Abstract

Nonspecific inflammation in the transplant microenvironment results in **β**-cell dysfunction and death influencing negatively graft outcome. MicroRNA (miRNA) expression and gene target regulation in transplanted islets are not yet well characterized. We evaluated the impact of inflammation on miRNA expression in transplanted rat islets. Islets exposed *in vitro* to proinflammatory cytokines and explanted syngeneic islet grafts were evaluated by miRNA arrays. A subset of 26 islet miRNAs was affected by inflammation both *in vivo* and *in vitro*. Induction of miRNAs was dependent on NF-*κ*B, a pathway linked with cytokine-mediated islet cell death. RT-PCR confirmed expression of 8 miRNAs. The association between these miRNAs and mRNA target-predicting algorithms in genome-wide RNA studies of **β**-cell inflammation identified 238 potential miRNA gene targets. Several genes were ontologically associated with regulation of insulin signaling and secretion, diabetes, and islet physiology. One of the most activated miRNAs was miR-21. Overexpression of miR-21 in insulin-secreting MIN6 cells downregulated endogenous expression of the tumor suppressor Pdcd4 and of Pclo, a Ca^2+^ sensor protein involved in insulin secretion. Bioinformatics identified both as potential targets. The integrated analysis of miRNA and mRNA expression profiles revealed potential targets that may identify molecular targets for therapeutic interventions.

## 1. Introduction

Inflammation involves both the innate and adaptive immune systems following infection or injury. Deregulation of this process leads to chronic inflammation, generating a pathological response that favors destruction of the tissue involved [1]. Inflammation is the common denominator of several pathological conditions, including type 1 and type 2 diabetes. It also contributes to immune rejection in transplantation.

A plethora of proinflammatory mediators has been associated with toxicity and impairment of *β*-cell function [2], including cytokines [3–6], hyperglycemia, and hyperlipidemia [7–9]. Inflammation plays key roles in islet engraftment and survival after transplantation. During the early posttransplant period, islet cells are exposed to noxious stimuli, activation of macrophages, local secretion of chemokines, tissue factor induction, and formation of reactive oxygen species (ROS) due to hypoxic conditions, all causing an impairment of engraftment and function [10–12]. The nonspecific inflammation generated in the transplant microenvironment triggers adaptive immune responses, negatively influencing graft survival [13, 14].

Emerging evidence shows that small noncoding gene products, miRNAs, negatively regulate gene expression posttranscriptionally [15, 16]. MiRNAs play a critical role in inflammatory diseases [17–19], in the vascular system [20] and diabetes [21–25]. In this study, we determined the expression signatures of miRNAs in islets exposed to proinflammatory conditions *in vitro* [2] or after transplantation. Locked Nucleic Acids-probe (LNA) MicroRNA hybridization arrays and significance analysis of microarray (SAM) identified miRNA subsets modulated by both experimental conditions. To identify genes that are directly targeted by these miRNAs, we performed bioinformatic analysis relating the miRNA expression profiles with genome-wide RNA (GWR) microarray studies focusing on inflammation of pancreatic *β*-cells [26, 27].

This approach may lead to the development of molecular therapies to alter expression of involved miRNAs and their specific targets, which may enhance preservation of *β*-cell function and survival after transplantation.

## 2. Material and Methods

### 2.1. Islet Isolation

Animal procedures were performed under protocols reviewed and approved by the University of Miami IACUC. Lewis rats (Harlan, Indianapolis, IN) of either sex were used as donors and recipients of islet cells. Islets were obtained by a mechanically enhanced enzymatic digestion using Liberase (Roche) followed by separation on discontinuous density gradients (Mediatech) [28]. After overnight culture at 37°C, 5% CO_2_ in supplemented CMRL-1066 medium (Gibco-Invitrogen), islet aliquots were prepared in non-treated tissue culture dishes for *in vitro* or transplantation experiments.

### 2.2. Islet Exposure to Inflammation *In Vitro*

 After overnight culture, isolated islets were exposed *in vitro* to a proinflammatory cytokine cocktail [29]. Briefly, recombinant human cytokines utilized in combination were as follows: interleukin-1-beta (IL-1*β*; 50 U/mL), interferon-gamma (IFN-*γ*; 100 U/mL), and tumor necrosis factor-alpha (TNF-*α*; 2,000 U/mL), all from R&D Systems. Islets were exposed to the treatment for 6 and 18 hours. In selected experiments aimed at dissecting the role of NF-*κ*B pathway in cytokine-induced islet miRNA expression, islets were pretreated for 2 hours with the NF-*κ*B inhibitor Bay11-7082 (5 uM), which inactivates I*κ*B-*α* phosphorylation, and then cultured with the cytokine cocktail for additional 6 hours. Untreated islets cultured in parallel were used as controls. At the end of the incubation period, islets were collected in saline solution, and then stored in RNALater until processed for molecular arrays.

### 2.3. Islet Transplantation and Graft Recovery

Islet aliquots (~3,000 IEQ each) were transplanted under the kidney capsule, as described [14]. After three days, islet grafts were collected by careful dissection from the renal subcapsular space and stored in RNALater for molecular arrays.

### 2.4. Overexpression of miR-21 in MIN6 Cells

The MIN6 cells were transfected with 200–400 nM mimic miR-21 (Dharmacon) or 200–400 nM irrelevant control using transfection reagent “Dharmafect” following the manufactures instructions. Mimic transfected cells and their controls were cultured 48 hours, harvested and subjected to qRT-PCR.

### 2.5. LNA-Oligonucleotide-Probes-Based Hybridization Arrays

RNA was labeled (Hy3 or Hy5 fluorescent dye) using the miRCURY LNA Array Power labeling kit (Exiqon). The labeled RNA molecules were hybridized to the miRCURY LNA Array slides (Exiqon) that contain capture probes for 279 rat microRNA genes complementary to mature miRNAs, registered in miR-Base Release 9.2. After hybridization, the chips underwent image acquisition (Scanner Axon model 4100A; Molecular Devices) and the data analyzed using GenePix Pro 6.0 image analysis software. Replicate hybridizations of the same control/experimental samples were performed utilizing the two-color ‘‘dye flip reversal method.” This experiment was repeated with 3 samples for a total of six hybridizations.

The averages of both hybridizations (Hy3/Hy5 and Hy5/Hy3) for three samples were analyzed by Significant Analysis of Microarray (SAM). Only miRNAs detected in both dye flip reversal were included in the analysis. SAM calculates *q*-values, a measure of significance based on False Discovery Rate concept for genome-wide association studies [30]. To increase the stringency of the analysis only samples with *q* = 0 were considered.

### 2.6. Quantitative RT-PCR

Total RNA was isolated from transplanted islets using the mirVana miRNA Isolation kit (Ambion). The isolated RNA can be used for miRNA as well as mRNA analysis. cDNA synthesis and PCR amplification were performed according to the manufacturer's protocol (Applied Biosystems). MiRNA profiling was performed using micro-fluidic cards TaqMan Low Density Array (TLDA, v1.0) for rodent miRNAs, which allow quantitative assessment of 365 miRNAs using the AB7900 instrument (Applied Biosystems). Quantification of miR-21 and mRNA was carried out in a 7500 Fast Real-time PCR system, utilizing TaqMan reagents (Applied Biosystems) using (RQ) values. RQ represents the fold changes of expression between control and treated samples, for example, nontransplanted islets versus transplanted islets. RQs were calculated with the Applied Biosystems SDS software. The number of amplification cycles, Ct, is normalized to endogenous control 18S rRNA for the TLDA, and beta-actin and snoRNA135 for mRNA and miR-21 assessments, respectively.

### 2.7. Semiquantitative RT-PCR Analysis of Pclo Splicing Versions

PCR was performed using the following primers: Pclo forward primer sequence TCCAAGGATATGCAGGTTCC is shared by both versions (V1 and V2, resp.) and spans between exons 19 and 20. The reverse primers are specific for each version and are as follows: ACGCTATACCCACTGCCAAC (V1) and TGAACATTAAGCTGCCATGC (V2).

## 3. Results

### 3.1. MicroRNA Expression in Islets after *In Vitro* Treatment with Proinflammatory Cytokines

Inflammation can be mimicked *in vitro* by exposing islets to proinflammatory cytokines. Specifically, IL-1*β* induces functional impairment and cell death in cultured islets [31], while TNF-*α* and IFN-*γ* enhance cytotoxicity [32]. The miRNA expression patterns in rat islets exposed to cytokine cocktail [IL-1*β* (50 U/mL), TNF-*α* (2000 U/mL), and IFN-*γ* (100 U/mL)] for either 6 or 18 hours (*n* = 3) were assessed by LNA-(locked nucleic acids) based microarray analysis, using Exiqon chips. We chose the LNA probes because of their accurate sequence discrimination and strong hybridization [33]. They are comparable to the emergent next-generation sequencing (NGS) high throughput miRNA profiling via RNA sequencing [34].

The maximum cytokine effect on miRNA profiles occurred 6 hours after treatment, while after 18 hours the effect was markedly reduced (data not shown). The results obtained from the miRNA microarrays (*n* = 3) were analyzed by SAM [30], adopting a *q* = 0. *Q*-values correspond to the *P* values adapted to the analysis of a large number of genes; *q* = 0 is the minimum false discovery rate and refers to the chance that a given miRNA is a false positive (fold changes greater than 2.0). We identified a pool of 64 miRNAs (Table 1). The NF-*κ*B pathway has a critical role in cytokine-induced islet cell death [35]. Therefore, we investigated if the cytokine-mediated induction of miRNAs was also dependent on NF-*κ*B activation in our study. Islets were pretreated for 2 hours with the NF-*κ*B inhibitor Bay 11-7082 that inactivates I*κ*B-*α* phosphorylation [36] and then cultured with the cytokine cocktail. The high throughput miRNA assay using the Exiqon platform with SAM showed that blocking of NF-*κ*B pathway caused significant reduction (on average more than 50%, range 24–72%) in the activation of most miRNAs tested (Table 2).

### 3.2. Islet miRNA Expression after Transplantation

Islet cells are exposed to multiple insults after transplantation [37, 38]. To investigate the effect of *in vivo* inflammatory milieu on the expression of islet miRNAs, we transplanted rat islets under the kidney capsule of syngeneic recipients, an experimental model well suited to study early inflammatory events. Since the implanted islets remain in a well defined mass under the kidney capsule, they can be easily retrieved with minimal contamination from surrounding tissue for molecular evaluation [39]. The grafts were retrieved. MiRNA expression on islet grafts explanted 3 days after transplant (*n* = 3) was analyzed using LNA (Exiqon) microarrays and subsequent SAM. Nontransplanted islets taken from the same isolation served as controls. Explanted grafts yielded a pattern of 31 miRNAs with altered expression: 26 of them were upregulated and 5 downregulated. PCR-based TaqMan Low Density Arrays confirmed the expression of 11 miRNAs. Comparing the miRNA patterns of expression in the *in vitro* and *in vivo* experiments, we retrieved a subset of 26 miRNAs commonly regulated under both experimental conditions, 24 were upregulated and 2 downregulated (Table 3). Eight miRNAs from the PCR-confirmed 11 miRNAs, are common to both *in vitro* and *in vivo* inflammation conditions; 7 upregulated (miR-21, miR-98, miR-27a, miR-143, let-7d, miR-126 and miR-22) and one (miR-129) downregulated (Table 3). 

### 3.3. Inflammation-Induced miRNAs and Identification of Their Potential Targets

MiRNAs downregulate their target mRNA levels causing inhibition of translation [40]. Furthermore, a recent study showed that destabilization of target mRNAs is the major reason for reduced protein expression mediated by miRNAs [41]. To identify potential miRNA targets, we integrated our high throughput miRNA expression data with previously published transcriptome studies. We expected that upregulated miRNAs would correspond to downregulated RNA targets and *vice versa*. Specifically we looked for inverse association between the expression of islet miRNAs modified by inflammation via both *in vivo* and *in vitro* conditions and the algorithm-predicted target genes found in a genome-wide mRNA (GWR) expression studies by Ortis et al. [27]. These authors studied the modification of genes in primary rat *β*-cells exposed* in vitro* to combination of the same cytokines we used in our study, namely IL-1*β* + IFN-*γ* or TNF-*α* + IFN-*γ* [27]. 

Two of the most commonly used computational miRNA predictive target programs are miRANDA and PicTar [42–44]. It has been shown that perfect “seed” pairing is important for miRNA target recognition and predictability. The “seed” is the sequence corresponding to nucleotides 2–7 of the miRNA's 5′region [45]. The predictions by PicTar have a higher degree of overlap because these algorithms are based on stringent “seed” pairing, unlike miRBase that employs moderate “seed” pairing [46]. It has been reported that prediction of targets did not improve by using overlapping algorithms [47]. For that reason, rather than selecting the common targets to both algorithms, we chose PicTar algorithm to match upregulated miRNAs (miR-21, miR-98, miR-27a, miR-143, let-7d, miR-126, miR-22) with downregulated putative targets in Ortis et al. and vice versa (Table 4). The PicTar algorithm identified 202 downregulated mRNAs which according to Ortis' study are potential targets for either of the above-mentioned upregulated miRNAs, and 36 upregulated mRNAs the possible targets for downregulated miR-129 (Table 4). From the 202 downregulated genes, 108 are targeted by one miRNA, and the rest are targeted by multiple miRNAs (data not shown). Interestingly, 30 genes are associated with regulation of insulin signal transduction pathways, development, and function of pancreatic islets, insulin secretion, and insulin resistance (Table 4). 

### 3.4. Regulation of Endogenous mRNA by miR-21

**Figure 1:**
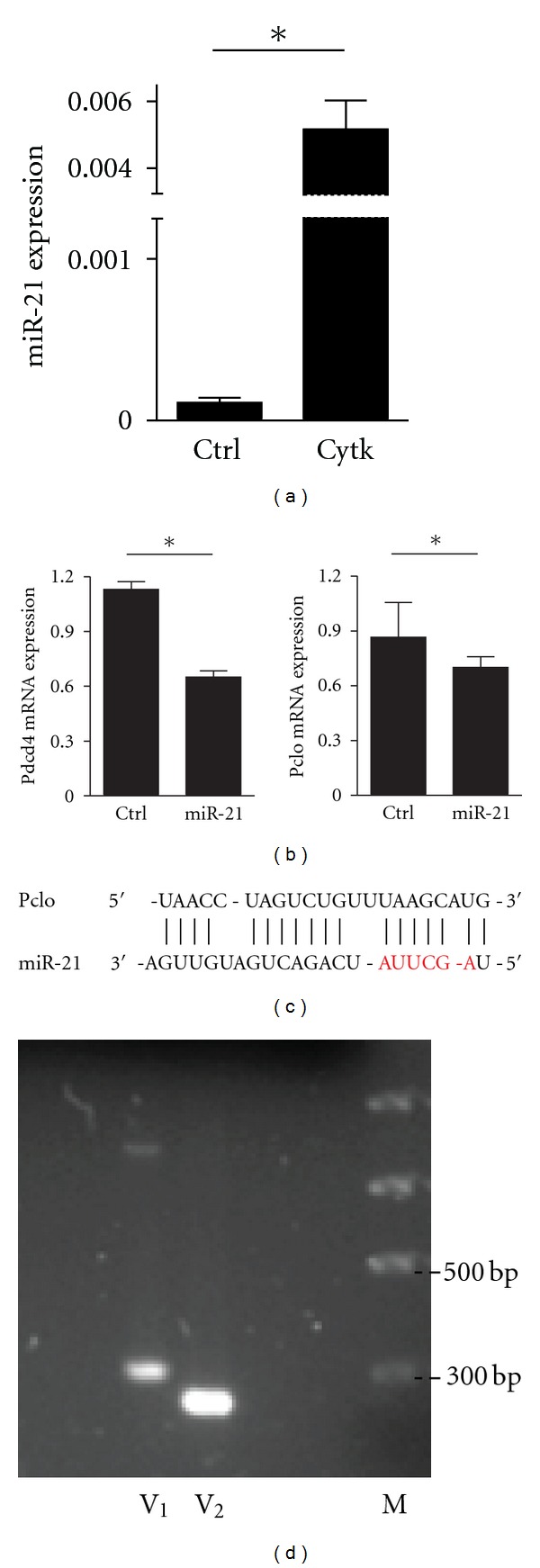
Overexpression of miR-21 regulates endogenous Pdcd4 and Pclo mRNAs. (a) MIN6 cells were treated 24 hours with cytokine cocktail IL-1*β* (50 U/mL), TNF-*α* (2000 U/mL), and IFN*γ* (100 U/mL). The expression of miR-21 was assessed by qRT-PCR. (b) Overexpression of miR-21 mimic (300 nM) for 48 hs inhibits the expression of endogenous Pdcd4 and Pclo mRNA. Experiments shown in (a) and (b) are expressed as mean ± SD (*n* = 5), *P < 0.05 (*t*-test, 2 tails). (c) miR-21 recognition site in the 3′UTR of Pclo splicing version 2. MiR-21 “seed” is shown in red font. (d) Semiquantitative RT-PCR shows expression of both Pclo versions (V1, V2) in MIN6 insulinoma cells.

To further test whether some of these 30 genes (Table 4) could be associated functionally with specific miRNAs, we studied selected miRNA overexpression effect on putative endogenous mRNA targets in the mouse insulin-secreting cell line MIN6. Since miR-21 was identified as the most reproducibly induced miRNA *in vitro* and *in vivo, *with the highest score “*d*” in SAM analysis (Table 3), we focused on this miRNA. Programmed cell death 4(Pdcd4) and Piccolo (Pclo) are miR-21 potential targets (Table 4). Pdcd4 is a tumor suppressor gene that inhibits neoplastic transformation, tumor progression, and translation. It has been identified as miR-21 target in several systems [84]. Pclo is a high molecular weight (550 kDa), multidomain protein functioning as a scaffold for proteins involved in synaptic vesicle endo- and exocytosis near their site of action. Pclo is proposed to function as Ca^2+^ sensor protein in cAMP insulin secretion in islets [50]. *In vitro* treatment of MIN6 cells with cytokines induced the expression of miR-21 (Figure 1(a)). Overexpression of miR-21 achieved by the addition of a mimic miR-21, but not of an irrelevant mimic miRNA (control), decreased the expression of both Pdcd4 and Pclo mRNAs in MIN6 cells (Figure 1(b)). It has been described that Pclo consists of two major splicing versions: V1 and V2 [85]. Their biological role is not currently known. Only V2 has an miR-21 recognition site in its 3′UTR. The miR-21: Pclo interaction site has a mismatch in the “seed” region; however, it displays a more extensive base pairing at the 3′ end of the miRNA (Figure 1(c)). Semiquantitative PCR analysis showed that Pclo V2 is predominant in MIN6 cells (Figure 1(d)). Therefore, we could confirm the specificity of miR-21 to induce downregulation of endogenous Pclo in *β*-cells. These results suggest that in pancreatic islets miR-21 targets both Pdcd4 and Pclo genes. 

## 4. Discussion

Islets of Langerhans are highly vascularized endocrine cell clusters located in the pancreas. The islet isolation process utilizes fragmentation of the gland to free the islets from the surrounding tissue, which results in a loss of vascular support. Consequently, the islets undergo hypoxic stress that persists until full revascularization in the recipient's microenvironment is completed, which may last several weeks [86]. Multiple factors, such as the duration of organ ischemia and the islet isolation process, contribute to activation of stress-induced signal transduction pathways and generation of inflammation mediators by islet cells [28]. Thus, islet cells participate actively in the initiation of local inflammation, which is further triggered by the transplant procedure. These responses may further amplify adaptive immunity responses after transplantation resulting in impairment of *β*-cell function and viability. Modulation of inflammatory responses in the early peritransplant period is associated with improved islet engraftment and function in both experimental and clinical settings. The purpose of our study was to identify islet microRNAs modulated *in vitro* and *in vivo* by inflammatory events. In the clinical settings, the islets are implanted into the hepatic portal system where they are exposed to blood, ischemia and activation of endothelium all contributing to the inflammatory reaction elicited in the transplant microenvironment. Unfortunately, it would be quite cumbersome to retrieve the graft from the liver for molecular analysis without introducing important biases (i.e., enzymatic digestion and purification to collect the islets otherwise randomly distributed into the liver parenchyma). Conversely, despite lacking key features of the intrahepatic site, the kidney subcapsular space allows the easy retrieval of the grafted tissue for molecular analysis with minimal manipulation. 

The molecular pathways involved in islet cell response to inflammation during the peritransplant period are yet to be fully understood. Transcriptome analysis of explanted islet grafts has revealed a key involvement of NF-*κ*B pathway as an initial adaptation response to the new microenvironment and the underlying tissue remodeling during the peritransplant period [87]. 

The emerging role of miRNAs as master regulators of gene expression has opened new avenues toward the thorough understanding of cellular responses under various physiological and pathological conditions. Indeed, herein we report that miRNAs expression is regulated by the inflammatory milieu generated in transplanted islets. We have identified a pool of 26 miRNAs commonly affected by inflammation both *in vivo* and *in vitro*, suggesting their association with the intrinsic basic molecular mechanism determining the fate of islet grafts in the early posttransplant period. 

Our study includes an integrated evaluation of miRNAs and mRNAs gene expression in insulin secreting cells along with functional studies identifying new targets for miRNAs activated by proinflammatory cytokines. Induction of miRNA transcription was partially dependent on activation of NF-*κ*B, a transcription factor with a critical role in *β*-cell apoptosis mediated by proinflammatory cytokines (Table 2) [35, 88]. The convergence of miRNAs and NF*κ*B signaling pathway has been recently established [89]. Using quantitative PCR-based high throughput analysis, we have confirmed upregulation of 7 (miR-21, miR-98, miR-27a, miR-143, let-7d, miR-126, and miR-22) and downregulation of 1 (miR-129) miRNAs out of the 26 activated miRNAs identified in our settings. The relatively low number of miRNAs confirmed by RT-PCR in our study might be due to the low reproducibility of miRNA profiling interplatforms [90]. This may also explain why miRNAs previously reported in islets upon *in vitro *cytokine exposure, such as miR-146 and miR-34a, were not confirmed in our study [25]. Some of the 8 miRNAs have been reported previously in studies related to islet physiology or diabetes. MiR-21 has a potential role in diabetic nephropathy [91]. Low plasma levels of miR-21 and miR-126 have been detected in patients with type 2 diabetes [92]. In agreement with our results, cytokines increased miR-21 expression in *β*-cells, while miR-21 downregulation conferred cytoprotection to islets exposed to IL-1*β* *in vitro* [25]. The expression patterns of miR-27a varied with hyperglycemia in the Gyoto-Kakizaki rat [5], and miRNA-143 overexpression inhibited insulin-stimulated AKT activation and resulted in impaired glucose metabolism [93]. 

Our results suggest that overexpression of miR-21 in MIN6 cells could regulate the expression of Pdcd4 and Pclo steady-state mRNA levels. The tumor suppressor proinflammatory protein Pdcd4 promotes activation of the transcription factor NF-*κ*B [94]. Downregulation of Pdcd4 by miR-21 has been associated with attenuation of cytotoxic effects of oxidative stress and ischemia-reperfusion in cardiomyocytes [95, 96], decreasing the proinflammatory effects of TLR4 signaling [94], and also preventing type 1 diabetes in rodents [49]. Furthermore, miR-21 targets the Pclo gene which acts as a Ca^2+^ sensor via formation of a cAMP-GEFII(Epac2)-Rim2 complex in PKA-independent cAMP insulin secretion. Pclo inhibition impairs cAMP insulin secretion [50]. Therefore, miR-21 has the ability to regulate genes such as Pclo and Pdcd4 that might affect *β*-cells in conflicting manner. On one hand, during inflammation miR-21 contributes to the impairment of islet cells function by interfering with insulin exocytosis via downregulation of Pclo. On the other hand, miR-21 could reduce cytokine-mediated apoptosis in *β*-cells via downregulation of Pdcd4. Divergent effects have been also reported in islets treated with cytokines for mRNAs, such as STAT1 and IRF-1 [97]. 

Collectively, our study and the results of previous reports regarding the effect of cytokines on gene expression in islet *β*-cells [25–27, 98] indicate that proinflammatory cytokines trigger a complex response resulting in modulation of the expression of islet mRNAs and miRNAs, which in some cases might affect the system in a seemingly contradictory fashion. In the context of transplantation, it is likely that the final outcome of the cytokine effect on islet cells depends on the combination of factors, such as intensity and duration of exposure, and initial quality of the islet graft (i.e., viability). 

In conclusion, we found a set of miRNAs that are regulated by inflammatory conditions in transplanted islets. In addition, the theoretical bioinformatics analysis identified potential genes that are directly regulated by these miRNAs. This information could be helpful for future studies of novel genes involved in inflammation-mediated *β*-cells dysfunction as well as for the development of new therapeutic applications. 

## Figures and Tables

**Table 1:** miRNAs expressed in islets treated *in vitro* with a cocktail of IL-1*β*, IFN-*γ*, and TNF-*α* were identified by SAM of global miRNAs. The score (*d*) represents value of the T-statistic; a higher score means a greater difference between the two groups. *q*-values correspond to the *P* values adapted to the analysis of a large number of genes. FI and SD are fold increase and standard deviation of hybridization values (cytokine versus control), respectively.

Upregulated miRNAs	(*d*)	*q* (%)	FI	SD		miRNA	(*d*)	*q* (%)	FI	SD
miR-143	6.7	0	7.5	3.5		miR-27a	4.5	0	6.2	4.2
miR-30e	6.0	0	15.3	11.4		let-7a	4.4	0	8.4	7.1
miR-182	6.0	0	9.1	4.8		miR-375	4.4	0	7.8	6.4
miR-96	6.0	0	13.6	9.4		miR-30a	4.4	0	5.3	3.3
miR-141	5.9	0	13.2	10.6		miR-19b	4.3	0	6.6	4.5
miR-24	5.7	0	9.2	6.2		let-7f	4.3	0	19.8	25.4
miR-29b	5.5	0	12.7	11.2		miR-16	4.1	0	4.7	2.9
miR-212	5.5	0	9.8	7.0		miR-29c	4.1	0	10.6	10.2
miR-7a	5.2	0	10.7	8.6		miR-103	4.1	0	3.0	0.9
miR-19a	5.2	0	3.9	1.3		miR-148b-3p	4.0	0	7.1	6.1
let-7i	5.2	0	13.7	9.6		miR-30d	3.9	0	4.1	2.3
miR-153	5.1	0	25.0	27.8		miR-301a	3.9	0	22.3	26.5
miR-22	5.1	0	4.7	1.9		let-7c	3.9	0	5.1	3.5
miR-27b	5.0	0	8.1	5.7		miR-23b	3.8	0	4.3	2.5
miR-21	5.0	0	10.2	6.5		miR-29a	3.7	0	5.8	4.5
miR-30b-5p	5.0	0	6.6	4.2		miR-23a	3.6	0	4.3	2.6
let-7d	4.9	0	9.6	7.7		miR-204	3.6	0	7.4	8.2
miR-30c	4.8	0	6.5	4.0		miR-541	2.9	0	13.1	16.0
miR-200a	4.8	0	13.0	11.5		miR-99b	2.9	0	13.1	1.3
miR-207	4.8	0	4.6	2.2		rno-let-7b	2.8	0	3.6	2.3
miR-183	4.8	0	4.7	2.0		miR-125b-5p	2.7	0	3.0	1.7
miR-127	4.8	0	7.6	3.8		miR-17/17-5p	2.7	0	9.9	14.2
miR-107	4.7	0	6.0	3.7		miR-194	2.5	0	2.9	1.8
miR-335	4.7	0	2.8	0.7		miR-200c	2.4	0	2.6	1.4
miR-101a	4.7	0	3.9	2.7		miR-125a-5p	2.4	0	2.6	1.5
miR-26a	4.7	0	6.5	4.5		miR-200b	2.3	0	2.7	1.5
miR-98	4.6	0	9.3	7.8		miR-340-5p	2.2	0	2.3	1.1
miR-7b	4.6	0	14.0	13.4		let-7e	2.0	0	2.3	1.1
miR-126	4.5	0	4.5	2.2		miR-337	2.0	0	2.0	2.3
miR-106b	4.5	0	4.0	1.5						

Downregulated miRNAs	(*d*)	*q* (%)	FI	SD						

miR-185	−2.8	0	4.0	3.3						
miR-129	−3.0	0	2.8	1.5						
miR-503	−3.2	0	4.1	3.2						
miR-370	−3.4	0	3.5	2.1						
miR-206	−4.3	0	2.6	0.7						

**Table 2:** miRNAs selected by SAM analysis of global miRNA expression in islets exposed *in vitro* to cytokines with or without inhibition of the NF-*κ*B pathway. Values are expressed as percentage of values obtained with NF-*κ*B inhibitor Bay 11-7082 plus cytokines versus cytokines alone.

miRNA	CTK + Bay	miRNA	CTK + Bay
	versus CTK (%)		versus CTK (%)
miR-206	72.8	miR-143	33.70
miR-17/17-5p	61.8	miR-24	33.71
miR-541	59.1	miR-370	33.71
miR-101a	57.6	miR-96	33.28
miR-503	54.0	miR-125a-5p	32.87
miR-106b	52.5	miR-200a	32.82
miR-194	51.0	miR-16	32.79
miR-204	50.9	miR-99b	32.70
miR-148b-3p	49.6	miR-26a	32.44
miR-19a	48.3	miR-200c	32.43
miR-19b	46.2	miR-337	32.39
miR-129	45.9	miR-30c	32.12
miR-301a	41.4	miR-7a	32.03
miR-30d	40.6	miR-23a	31.92
miR-127	40.2	miR-27a	31.91
miR-335	39.4	miR-30b-5p	31.81
rno-let-7f	39.4	miR-200b	31.67
miR-183	38.5	miR-125b-5p	31.63
miR-212	38.4	miR-375	30.78
rno-let-7e	37.7	rno-let-7b	30.71
miR-22	36.9	miR-27b	30.63
miR-29a	36.3	rno-let-7d	30.60
miR-98	36.1	rno-let-7c	30.57
miR-23b	35.8	rno-let-7a	30.31
miR-185	35.7	miR-153	29.32
miR-182	35.3	miR-30e	29.21
miR-207	35.3	miR-141	29.20
miR-340-5p	35.3	miR-29b	28.11
miR-107	34.8	miR-30a	27.84
miR-126	34.3	rno-let-7i	27.65
miR-7b	33.9	miR-21	25.75
miR-103	33.8	miR-29c	24.66

**Table 3:** SAM of miRNAs expressed in transplanted syngeneic islets collected 3 days after implantation. MiRNA expression in transplanted islets was compared to control isolated islets. 26 common miRNAs that displayed altered expression *in vivo* and *in vitro* (cytokine treated islets) are shown in ****bold **** font. MiRNA confirmed by PCR are marked with an X in the last column. FI and SD are fold increase and standard deviation of hybridization values (transplanted islets versus control islets), respectively.

Upregulated miRNAs	(*d*)	*q*-(%)	FI	SD	PCR
***miR-21***	7.75	0.00	26.58	13.21	X
***miR-98***	4.40	0.00	7.94	3.60	X
***miR-212***	4.33	0.00	51.61	43.25	
***miR-27a***	4.15	0.00	19.56	14.07	X
***rno-let-7i***	4.13	0.00	16.16	15.27	
***miR-207***	4.01	0.00	20.62	15.18	
***miR-143***	3.94	0.00	9.12	6.07	X
***rno-let-7a***	3.89	0.00	13.03	9.55	
***rno-let-7d***	3.89	0.00	7.20	3.30	X
***miR-126***	3.83	0.00	41.18	60.44	X
miR-195	3.68	0.00	8.12	5.00	X
***miR-22***	3.66	0.00	10.14	8.45	X
***miR-27b***	3.54	0.00	10.57	6.85	
***miR-107***	3.49	0.00	8.16	5.90	
***miR-99b***	3.38	0.00	5.01	2.15	
miR-214	3.34	0.00	10.52	7.15	X
***miR-125b-5p***	3.33	0.00	8.13	4.95	
***rno-let-7e***	3.20	0.00	3.76	1.37	
***miR-23a***	3.17	0.00	9.24	8.21	
***rno-let-7b***	2.95	0.00	5.27	3.79	
***miR-24***	2.93	0.00	7.61	6.34	
***miR-30e***	2.91	0.00	7.77	4.92	
***miR-23b***	2.87	0.00	7.37	6.20	
***miR-26a***	2.80	0.00	5.93	3.69	
***miR-29c***	2.77	0.00	6.97	4.10	
***miR-375***	2.74	0.00	6.04	3.89	

Downregulated miRNAs	(*d*)	*q*-(%)	FI	SD	PCR

miR-542-5p	−6.03	0.00	19.82	11.24	
***miR-129***	−5.09	0.00	25.58	25.01	X
miR-326	−3.03	0.00	5.89	4.12	X
***miR-370***	−2.85	0.00	4.78	1.87	
miR-484	−2.55	0.00	11.70	2.74	

**Table 4:** Genes identified as potential miRNA targets by PicTar algorithm. Genes in ****bold **** font are associated with regulation of insulin signaling and secretion, diabetes, and islet physiology.

miRNA	Genes				Description and function
miR-21↑	Acbd5	Crebl2	Xkr6	***Pdcd4***	Program cell death 4. Pdcd4 is a major transcript in *in vivo* pancreatic islet neogenesis [48]. Pdcd4-deficient NOD mice do not develop diabetes [49].
	Arglu1	Mrpl49	Zadh2		
	Arhgap24	Rqcd1	***Pclo***		Piccolo, presynaptic cytomatrix protein. A Ca^2+^ sensor protein important in cAMP insulin secretion [50].

miR-98↑	Abcb9	Dnajc1	Msi2	Zfp462	
	Aldh6a1	Dusp7	Nlk	***Ccnd1***	Cyclin D1. Enhances human beta-cell replication and function *in vivo* [51].
	Anapc5	Eif4g2	Npepl1	***Ghr***	Growth hormone receptor. Essential for maintaining pancreatic islet size and normal insulin sensitivity and glucose homeostasis [52].
	Asap1	Elovl4	Ppapdc2	***Gtf2i***	General transcription factor II. Assists to overcome various insults and to sustain pancreatic beta-cell function [53].
	Brd3	Frmd5	Robo2	***Isl1***	Insulin gene enhancer protein ISL-1. Role in endocrine pancreatic development [54]. Reduction in Isl1 expression results in the impairment of insulin expression [55].
	Btg2	Gnptab	Rufy3	***Pbx1***	Pre-B-cell leukemia transcription factor 1. Development and function of pancreatic islets [56].
	Bzw1	Golt1b	Sbk1	***Pbx2***	Pbx2-pre-B-cell leukemia transcription factor 2. Pancreatic development [57].
	Cnot2	Kif2a	Son	***Ppargc1b***	Peroxisome-proliferative-activated receptor, gamma, coactivator 1 beta. Effect in insulin resistance and T2D [58].
	Coil	Med14	Trib2	***Rgs16***	Regulator of G-protein signaling 16. Control aspects of islet progenitor cell activation, differentiation, and beta-cell expansion in embryos and metabolically stressed adults [59].
	Dhx57	Mobkl3	Ubfd1	***Vsnl1***	Visinin-like protein1. A Ca^2+^ sensor protein that regulates insulin secretion [60].

miR-27a↑	Abcb9	H3f3b	Nlk	Wnk1	
	Ank3	Hmgcr	Obfc2a	Ypel3	
	Appbp2	Kbtbd8	Pank1	Ywhab	
	Arglu1	Kcnk2	Pde10a	Zadh2	
	Asph	Lpcat1	Phb	Zfp148	
	Btg2	Marcks	Pskh1	Zfp462	
	Cdc25b	Med14	Rcan2	Zhx1	
	Cdh11	Mrps14	Rpn2	***Abca1***	ATP-binding cassette, subfamily A, member 1. Influences insulin secretion and glucose homeostasis [61, 62].
	Dcx	Msi2	Sbk1	***Acly***	ATP citrate lyase. Protects against free-fatty-acid-mediated apotosis of beta-cells [63].
	Elmo1	Mycbp	Sgpp1	***Bnip3l***	BCL2/adenovirus E1B interacting protein 3-like. Critical mediator of *β* cell apoptosis and programmed necrosis in Pdx1-deficient diabetes [64].
	Fbxo33	Nap1l3	Smarca1	***Irs1***	Insulin receptor substrate 1. Islets from IRS-1 knockout mice exhibit marked insulin secretory defects and reduced insulin expression [65].
	Fubp3	Ncald	Stx16	***Isl1***	Described above.
	Galnt5	Necap1	Tardbp	***Map3k12***	Mitogen-activated protein kinase kinase kinase 12. Activation of Map3k12 by cyclosporin A induces beta-cell apoptosis in posttransplant diabetes [66].
	Golt1b	Nedd4	Tmtc2	***Myt1***	Myelin transcription factor. Myt1 is involved in proper endocrine differentiation and function [67].
	Gse1	Neo1	Ubfd1	***Snap25***	Synaptosomal-associated protein 25. Role in exocytotic vesicle recycling and granule exocytosis in pancreatic beta-cells [68].
	Gtf2i	Nf1	Usp9x	***Sv2a***	Synaptic vesicle protein 2. Role in Ca^2+^-dependent function in insulin exocytosis [69].

miR-143↑	Ash1l	Frmd5	Necap1	Vapb	
	Atp6v1a	Josd1	Ntrk2	Zfp148	
	Cbfb	Marcks	Ppp4r2		
	Cnnm3	Msi2	Tsc22d3		

let-7d↑	Abcb9	Elovl4	Nlk	***Gnaq***	Guanine nucleotide binding protein. Inactivation of Gnaq resulted in impaired glucose tolerance and insulin secretion in mice [70].
	Bzw1	Frmd5	Pbx1	***Isl1***	Described above.
	Cnih	Gnptab	Ppapdc2	***Pbx2***	Described above.
	Coil	Golt1b	Pskh1	***Rgs16***	Described above.
	Dcaf8	Ip6k2	Robo2	***Rhob***	Ras homolog gene family, member B. RhoB is an early-response gene whose expression is elevated by cellular stresses. It is important for the induction of *β*-cell loss [71].
	Dhx57	Magt1	Rufy3		
	Ebag9	Med14	Sdc2		
	Eif4g2	Myh10	Slc24a2		

miR-126↑	Ahcyl2	Ergic2	Nf1	Slc7a5	
	Atp2b1	Fbxo33	Ppm1b	Spred1	
	Atrn	Fyttd1	Ppp1r10	Wdr47	
	Bcl2l2	Gnaq	Ppp4r2	Zadh2	
	Bet1	Gria2	Psmc6	***Acsl6***	acyl-CoA synthetase long-chain family member 6. It mediates the postive effect of dehydroepiandrosterone (DHEAS) on insulin secretion [72].
	Bzw1	Irs1	Rbbp6	***Eif4a2***	Eukaryotic translation initiation factor 4A2. EIF4A2 is a positional candidate gene linked to type 2 diabetes. It is downregulated by glucose in INS1 cells [73].
	Efnb1	Necap1	Rit2		

miR-22↑	Anapc5	Ntrk2	Tmem50b	***Csnk2a1***	Casein kinase 1. Regulation of insulin production in islets [74].
	Calm3	Nudt4	Trib2	***Etv1***	Ets variant 1. It is regulated by Nkx2.2 during the major wave of pancreatic endocrine and exocrine cell differentiation [75].
	H3f3b	Ptprd	Vezf1	***Neurod1***	Neurogenic differentiation 1. Conversion of pancreatic progenitor cells into endocrine cells [76]. Contribute to beta-cell-specific and glucose-responsive insulin gene transcription [77].
	Map3k12	Rgp1	Wasf1		
	Necap1	Sv2a	Wnk1		

miR-129↓	Ash1l	H3f3b	Rab5b	Vps26a	
	Azin1	Hsph1	Rybp	Wee1	
	Bzw1	Itm2b	Sgms1	Zbtb44	
	Ctdspl2	Jag1	Slain2	Zfand3	
	Cxxc5	Kpna4	Slc6a6	Zfp36l1	
	Eif3j	Magi3	Smndc1	***Crtc2***	CREB-regulated transcription coactivator 2. Crtc2 is a coactivator of the cAMP response element-binding [78, 79].
	Etv5	Pkia	Sp1	***Mark2***	MAP/microtubule affinity-regulating kinase 2. Block the Creb:Crtc2 interaction [80].
	Fbxw2	Ppp1r14c	Sp3	***Pten***	Phosphatase and tensin homology. Deletion of Pten, a negative regulator of the P13K pathway, leads to increased *β*-cell mass and function [81, 82].
	Gmfb	Rab21	Tiparp	***Tiam1***	T-cell lymphoma invasion and metastasis 1. Tiam1 negatively affect glucose-stimulated insulin secretion [83].
